# Characterization of Proprioceptive System Dynamics in Behaving *Drosophila* Larvae Using High-Speed Volumetric Microscopy

**DOI:** 10.1016/j.cub.2019.01.060

**Published:** 2019-03-07

**Authors:** Rebecca D. Vaadia, Wenze Li, Venkatakaushik Voleti, Aditi Singhania, Elizabeth M.C. Hillman, Wesley B. Grueber

**Affiliations:** 1 Grueber Laboratory, Mortimer B. Zuckerman Mind Brain Behavior Institute, Columbia University, New York, NY 10027, USA; 2 Department of Neuroscience, Columbia University Irving Medical Center, New York, NY 10032, USA; 3 Laboratory for Functional Optical Imaging, Mortimer B. Zuckerman Mind Brain Behavior Institute, Columbia University, New York, NY 10027, USA; 4 Department of Electrical Engineering, Columbia University, New York, NY 10027, USA; 5 Department of Biomedical Engineering, Columbia University, New York, NY 10027, USA; 6 Department of Genetics and Development, Columbia University Irving Medical Center, New York, NY 10032, USA; 7 Department of Radiology, Columbia University Irving Medical Center, New York, NY 10032, USA; 8 Department of Physiology and Cellular Biophysics, Columbia University Irving Medical Center, New York, NY 10032, USA; 9 Kavli Institute for Brain Science, Columbia University, New York, NY 10032, USA; 11 Lead Contact

## Abstract

Proprioceptors provide feedback about body position that is essential for coordinated movement. Proprioceptive sensing of the position of rigid joints has been described in detail in several systems; however, it is not known how animals with a flexible skeleton encode their body positions. Understanding how diverse larval body positions are dynamically encoded requires knowledge of proprioceptor activity patterns *in vivo* during natural movement. Here we used high-speed volumetric swept confocally aligned planar excitation (SCAPE) microscopy in crawling *Drosophila* larvae to simultaneously track the position, deformation, and intracellular calcium activity of their multidendritic proprioceptors. Most proprioceptive neurons were found to activate during segment contraction, although one subtype was activated by extension. During cycles of segment contraction and extension, different proprioceptor types exhibited sequential activity, providing a continuum of position encoding during all phases of crawling. This sequential activity was related to the dynamics of each neuron’s terminal processes, and could endow each proprioceptor with a specific role in monitoring different aspects of body-wall deformation. We demonstrate this deformation encoding both during progression of contraction waves during locomotion as well as during less stereotyped, asymmetric exploration behavior. Our results provide powerful new insights into the body-wide neuronal dynamics of the proprioceptive system in crawling *Drosophila,* and demonstrate the utility of our SCAPE microscopy approach for characterization of neural encoding throughout the nervous system of a freely behaving animal.

## In Brief

Vaadia, Li et al. use high-speed 3D SCAPE microscopy to monitor proprioceptor morphology and calcium dynamics in behaving *Drosophila* larvae. Neural activity is coincident with dendritic deformations, and different subtypes exhibit sequential onset of activity during crawling. Analysis during non-stereotyped exploration shows proprioceptor versatility.

## INTRODUCTION

Monitoring neural activity in freely behaving animals is a key step toward understanding how sensory activity is transformed into action [1–3]. Small-invertebrate model systems with well-described sensory systems and complete or near-complete connectomes, such as *C. elegans* and *Drosophila* larvae, are ideal systems in which to uncover fundamental principles of sensorimotor integration. Light-sheet, confocal, and two-photon microscopy can capture neuronal calcium activity in isolated *Drosophila* brains or immobilized preparations [4–8]. However, these methods have been unable to provide volumetric imaging at sufficient speeds, in unrestrained samples, to enable extended imaging of body-wide neural activity in behaving animals.

**Figure 1.**
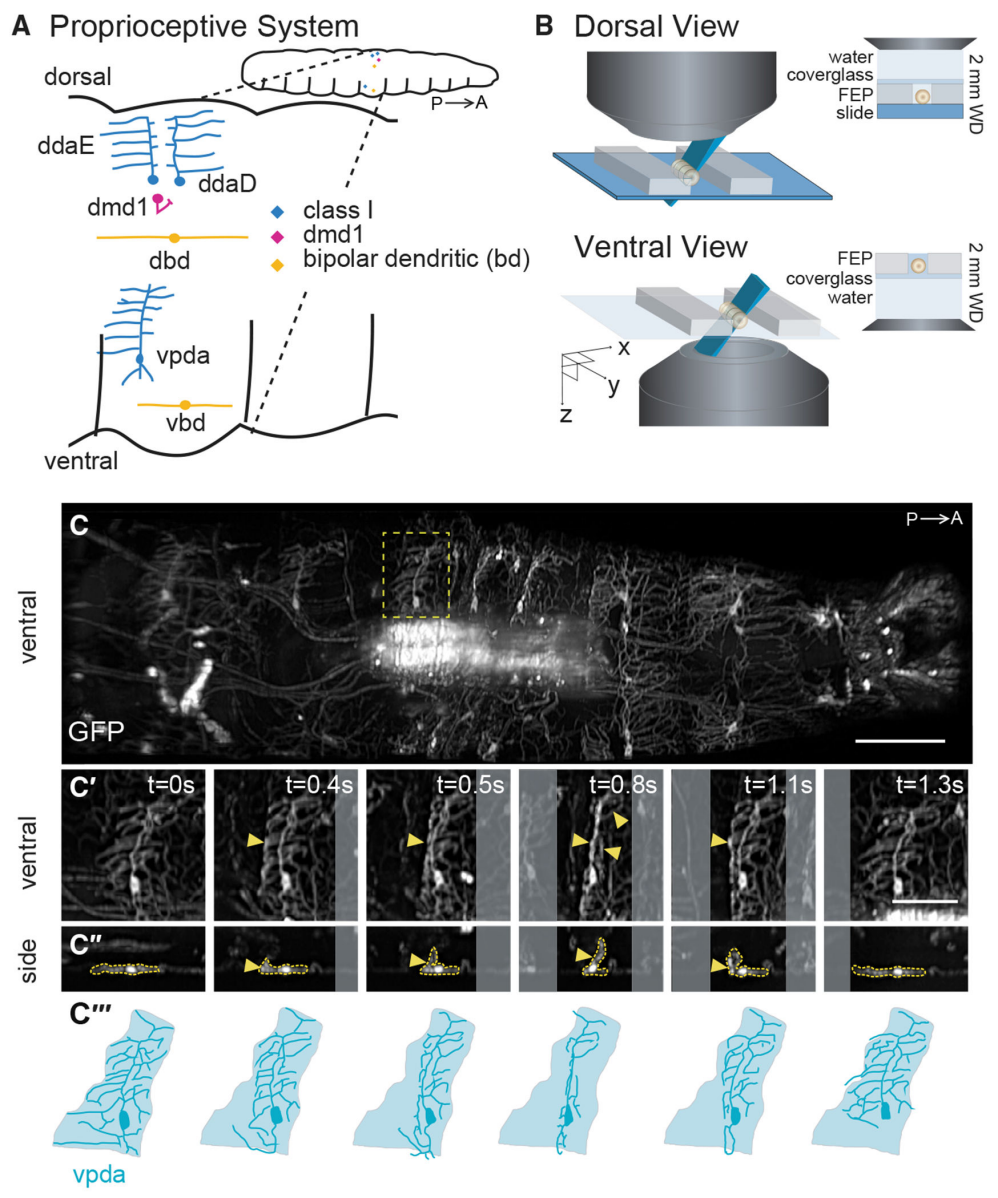
SCAPE Imaging of Proprioceptor Dendrite Motion Dynamics in Crawling Larvae (A) Schematic of the larval proprioceptive system. P, posterior; A, anterior. (B) Schematic of the larval imaging platform for SCAPE microscopy (see STAR Methods). (C) SCAPE imaging of a *221-Gal4*, *UAS-CD4tdGFP* larva ventral side during crawling. Maximum-intensity projection (MIP) over a 95-μm-depth range from a 160-μm-deep volume (to exclude gut auto-fluorescence, square root grayscale; see STAR Methods). See Video S1. The yellow box indicates the neuron examined in (C′)–(C‴). (C′ and C″) vpda in ventral view (C′) and side view (C″) in successive time-lapse frames during forward crawling. vpda dendritic arbors and cell body are outlined in yellow. Other arbors are from class IV neurons. (C‴) Tracing of neurons in (C′). Shaded areas represent dendritic field territory before folding. Posterior is to the left for all images. Scale bars, 100 μm (C) and 50 μm (C′ and C″).

Multispectral, high-speed, volumetric swept confocally aligned planar excitation (SCAPE) microscopy is capable of characterizing tissue and cellular dynamics in live behaving animals [9, 10]. We have applied this imaging technology to characterize the dynamics of multidendritic (md) neuron activity in crawling *Drosophila* larvae. Multifunctional md neurons are located just under the larval body wall and extend sensory dendrites along internal structures and the epidermis [11–16]. A subset of six of these md neurons (Figure 1A) extend axons to more dorsal neuropil regions important for motor control, suggesting that they are proprioceptors that provide feedback on body position [15, 17–19]. This feedback is thought to be particularly important during crawling, which involves periodic strides driven by peristaltic waves of muscle contractions along the body [20]. However, studies investigating the activity of these sensors have been limited to dissected preparations: imaging of axon terminals in an isolated central nervous system (CNS) suggests that at least some of these neurons are active during muscle contraction [21], whereas an electrophysiology study has shown activity in one cell type in response to stretch in a dissected preparation [22]. Studies that disabled all or some of these six neurons observed significantly slowed crawling, suggesting that these cells are proprioceptors that provide a segment contraction “mission accomplished” signal that promotes progression of the peristaltic wave [14]. These behavioral studies concluded that this set of neurons have partially redundant functions during crawling, because silencing different subsets caused similar behavioral deficits, whereas silencing both subsets had a more severe effect. However, the diverse dendrite morphologies and positions of these proprioceptor neurons [13, 15] suggest that each is likely to have distinct sensitivities and functions. Identifying the specific roles of each cell type is not possible without measuring the system’s dynamic activity patterns during natural movements to examine the synergies and dynamic encoding properties of the larval proprioceptive circuit.

Here, we characterized the spatiotemporal and functional dynamics of this set of *Drosophila* md proprioceptors by imaging neurons co-expressing GCaMP and tdTomato using SCAPE microscopy, with subsequent dynamic tracking and ratiometric calcium signal extraction. Characterization of the real-time dynamics of segment contraction and extension during crawling and exploratory head movements revealed that proprioceptors increased their calcium levels in synchrony with deformation of their dendrites. These cells provide a striking sequence of signaling during stereotyped forward crawling, suggesting an elegant continuum of sensing during movement. Furthermore, analysis of sensory responses during non-stereotyped exploration revealed a consistent relationship between activity patterns and more complex, asymmetric segment deformations. These activity patterns were found to be interpretable, via a simple linear combinatorial code, as separable representations of simultaneous turning and retracting movements. Our results provide valuable new input for models of how movements are controlled via feedback in the context of the larval connectome, and also demonstrate a new approach for characterization of body-wide neuronal dynamics in behaving *Drosophila*.

## RESULTS

### SCAPE Microscopy Allows 3D Imaging of Dendritic Deformations in Behaving Larvae

To begin to characterize proprioceptor dynamics as larvae crawl, we focused on the ventral posterior dendritic arborization (vpda) class I neuron (Figure 1A). Class I neurons spread sensory dendrites along the body-wall epidermis, suggesting that these cells may detect cuticle folding. To investigate how vpda sensory terminals deform during crawling, we first characterized dendrite dynamics using high-speed volumetric SCAPE microscopy [9, 10] at 10 volumes per s (VPS) as a larva crawled within a linear channel (Figures 1B and 1C). To achieve this imaging, we made numerous improvements to our original SCAPE microscopy system, including substantially improving spatial resolution to permit individual dendrites to be clearly resolved in 3D at high speed in the freely moving larva. We also increased the field of view to over 1 mm and made it sufficiently uniform to capture the entire crawling larva (see STAR Methods).

During forward crawling, peristaltic muscle contractions move from posterior to anterior along the animal [20]. vpda proprioceptors expressing GFP as a static marker showed repeated folding and extension: folding as a peristaltic wave entered a segment, and extending as the wave moved to anterior segments (Figures 1C–1C‴; Video S1). Viewed in cross-section, vpda dendrite tips flexed from distal to proximal, eventually angling at approximately 90° during each peristaltic contraction (Figure 1C″). Because class I dendrites are positioned along the basal surface of the epidermis [23], vpda dendrite dynamics most likely reflects body-wall dynamics. Therefore, our data indicate that vpda dendrites are positioned to respond to repeated contraction and extension of the body wall that occurs during crawling.

### SCAPE Microscopy of Neuronal Calcium Activity Dynamics in Behaving Larvae

Next, we sought to reveal whether and how the activity of these neurons changes as the dendrites fold during segment contraction. If vpda neurons indeed function as proprioceptors, we should be able to detect activity in these cells during locomotion. We built a dual-expression line of larvae to label targeted proprioceptive cells with both calcium-sensitive GCaMP (green) and static tdTomato (red). To acquire SCAPE microscopy data in this model, we optimized parallel dual-color imaging and developed a tracking algorithm that localizes the cell bodies via their static red fluorescence. These tracked cells were then used as fiducials for quantification of movement and behavior, as well as to extract and ratiometrically correct simultaneously recorded GCaMP fluorescence from the same cells.

**Figure 2.**
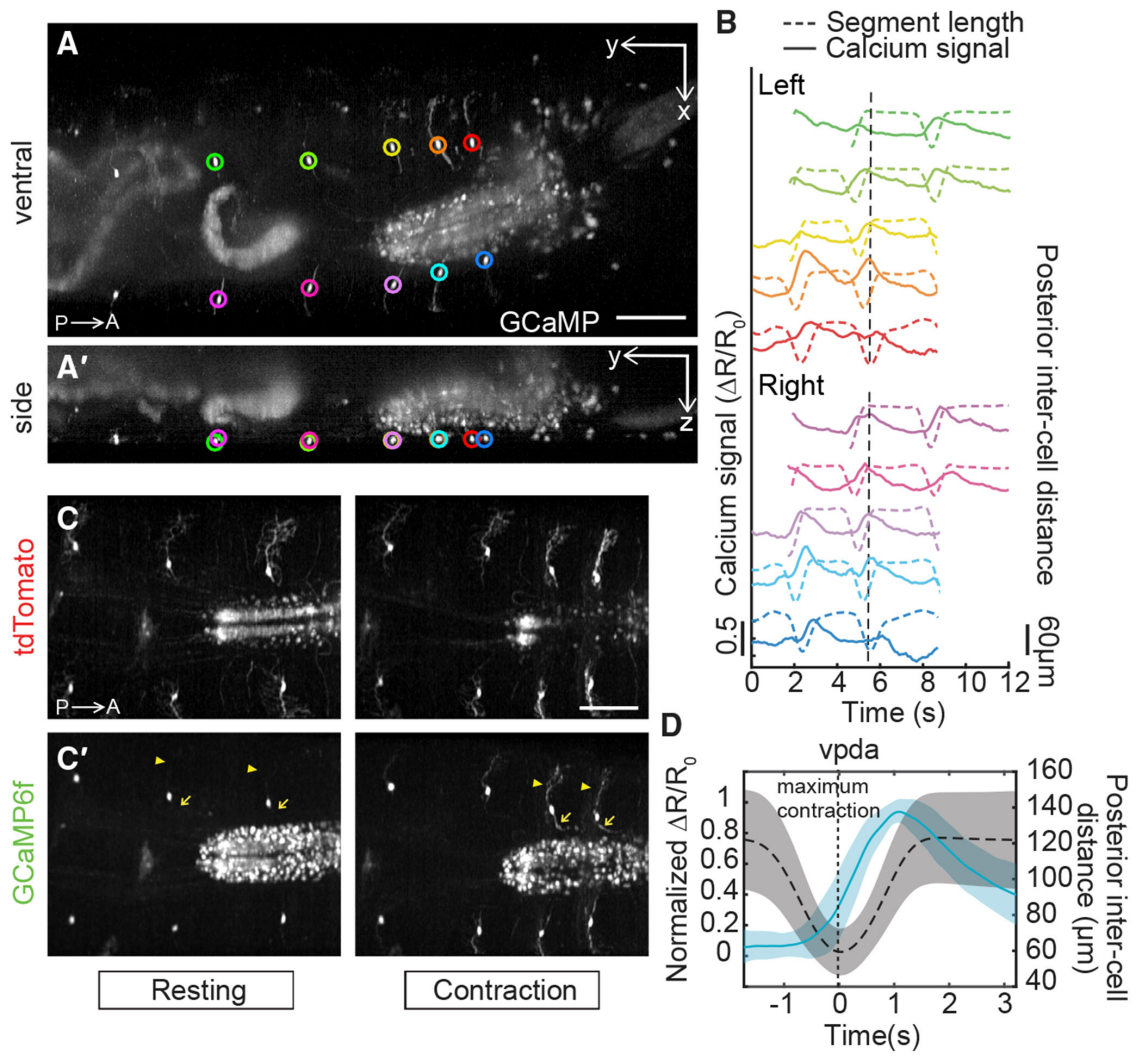
Dual-Color SCAPE Imaging of Proprioceptor Activity Dynamics in Crawling Larvae (A and A′) SCAPE imaging of a *410-Gal4*, *20XUAS-IVS-GCaMP6f* (×2), *UAS-CD4tdTomato* larva, ventral side, during forward crawling. Images are MIP of a full 168-μm-deep imaging volume (square root grayscale). (A) shows a ventral view (x-y) and (A′) shows a side view (y-z). GCaMP signal was extracted from the segmentally repeated vpda neurons indicated by circles. See Video S2. (B) vpda soma response for the neurons tracked in (A). The distance between the measured neuron and the posterior neuron (posterior inter-cell distance) is plotted in dashed lines, and the GCaMP6f response is plotted in solid lines (quantified as the fractional change in the green/red fluorescence ratio ΔR/R_0_; see STAR Methods and Figure S1). The dotted vertical line refers to the time point shown in (A). (C and C′) Representative tdTomato (C) and GCaMP6f (C′) images showing increased activity in dendrites (arrowhead) and axons (arrow) during contraction. Images are cropped to show region of interest and are MIP of a 70-μm-depth range from a 160-μm-deep volume (square root grayscale). See Video S3. (D) Mean calcium response (solid line) ± SD (ribbon) of vpda soma (3 animals, n = 22 cells [8, 10, and 4], 26 events [9, 13, and 4], respectively) during segment contraction, represented by mean posterior inter-cell distance (dashed line) ± SD (ribbon). Maximal segment contraction is set at “t = 0 s” for each event. ΔR/R_0_ amplitude was normalized for each event. Posterior is to the left for all images. Scale bars, 100 μm.

We used the inter-cell distance between the measured neuron and a homologous neuron in the posterior or anterior segment as our measure of segment contraction and extension. We observed consistent rises in GCaMP fluorescence in vpda neurons during each segment contraction (Figure 2). Calcium signals subsided as the peristaltic wave progressed to adjacent anterior segments (Figures 2B and 2D; Videos S2 and S3). Dynamic calcium responses were also visible in dendritic arbors and axons (Figures 2C and 2C′, arrowheads and arrows). Note that because of variability in calcium signal amplitude across contraction events (Figure 2B), signals were normalized for the averages shown in Figure 2D (see STAR Methods).

As a control, an additional larva line co-expressing GFP and tdTomato in vpda neurons was imaged using SCAPE during crawling. Applying the same tracking and ratiometric correction as for GCaMP, we observed insignificant changes in ratiometrically corrected GFP signal during crawling (Figure S1). Taken together, our data indicate that vpda neurons respond to body-wall folding during segment contraction.

### Monitoring of Different Proprioceptive Cell Types Reveals Distinct Activity Patterns

Having established this pipeline for cell characterization, SCAPE was then used to monitor the physical and functional dynamics of the remaining proprioceptive cell types, each of which has unique dendrite morphologies and positions (Figure 1A). Two additional class I neurons besides vpda project secondary dendrites along the dorsal side of the body wall (dorsal dendritic arborization neuron D [ddaD] anteriorly and dorsal dendritic arborization neuron E [ddaE] posteriorly; Figure 1A) [13]. These neurons are poised to detect cuticle folding on the dorsal side of the animal. In addition, dorsal and ventral bipolar dendrite md neurons (dbd and vbd, respectively) extend in an anterior-posterior direction, and it is known that at least dbd extends along internal connective tissue [12]. By contrast, neuron dorsal multidendritic neuron 1 (dmd1) extends an atypical thick dendrite from the body wall to the internal intersegmental nerve (ISN) [15], which lies along the muscle layer, suggesting that this proprioceptor could be poised to detect muscle dynamics.

**Figure 3.**
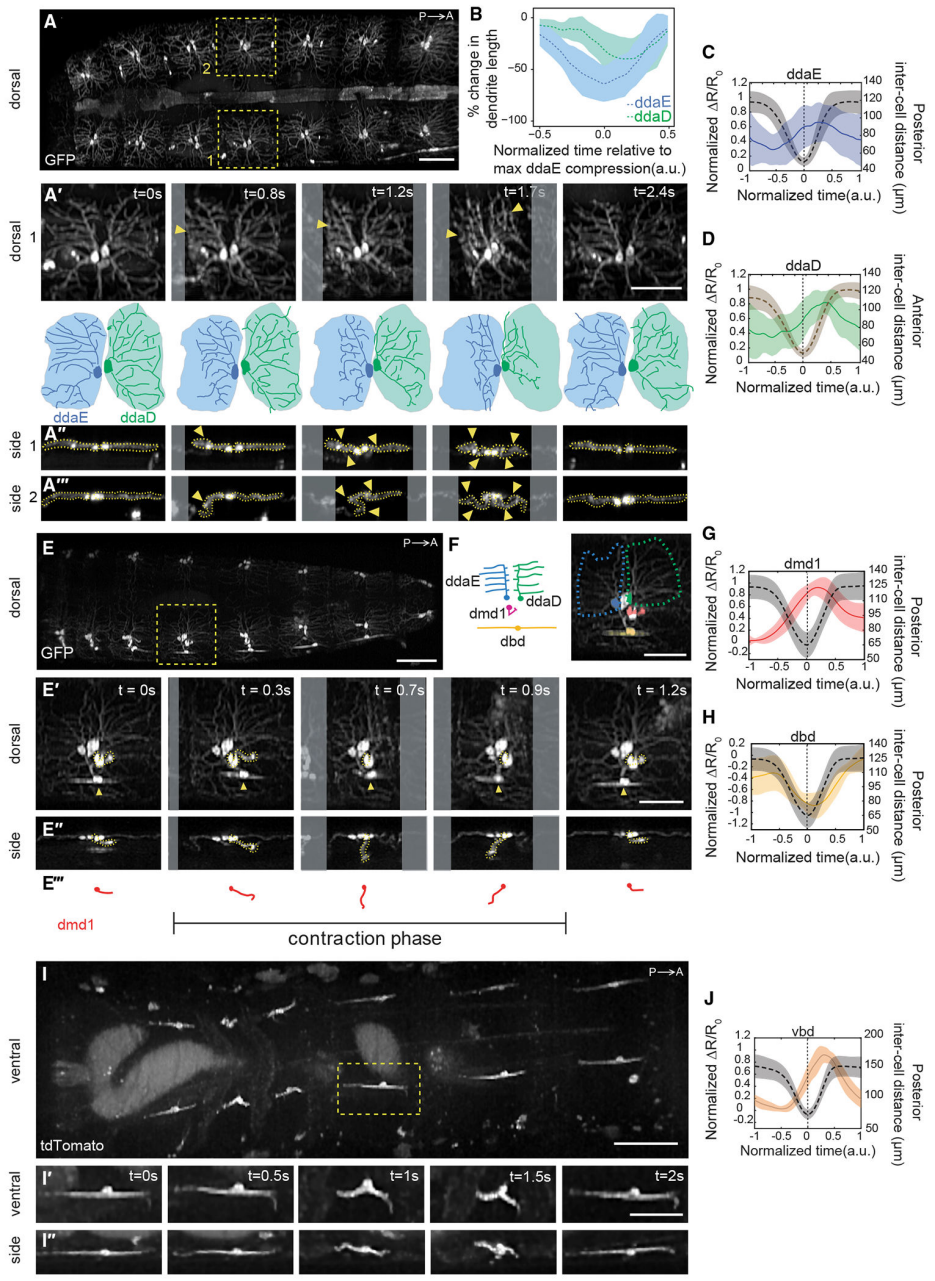
Proprioceptors with Diverse Morphologies Show Distinct Activity Patterns during Forward Crawling (A) Single frame from a SCAPE movie of a larva during crawling with ddaD and ddaE neurons visible on the dorsal side. Neurons were labeled using *221-Gal4, UAS-CD4tdGFP*. Yellow boxes indicate neurons examined in the time-lapse sequences below. (A′) Top: enlarged dorsal view of right-side neurons (1). Arrowheads indicate regions of dendrite folding. Bottom: tracing of neurons (top). Shaded areas represent dendritic field territory before folding. (A″ and A‴) Side view of right-side neuron (1) (A″) and left-side neuron (2) (A‴) during segment contraction. Arrowheads indicate dendritic folding. (B) Mean (± SD) percent change in dendritic length along the anteroposterior axis (a measure of dendritic folding) of ddaD (green; n = 10 cells) and ddaE (blue; n = 10 cells) from *221-Gal4, UAS-CD4tdGFP* (2 animals) and *410-Gal4, UAS-CD4tdtomato* (2 animals) during forward crawling. (See Figure S3 for changes to dendrite folding and activity in compressed conditions.) (C and D) Mean (± SD) calcium response (solid line) of ddaE (C) and ddaD (D) soma during segment contraction (quantified as the fractional change in the GCaMP/tdTomato fluorescence ratio ΔR/R_0_; see STAR Methods). Mean (± SD) inter-cell distance is plotted with a dashed line. Maximal contraction is set at t = 0 s for each event. In (D), we plotted anterior inter-cell distance between the measured neuron and the homologous neuron in the anterior segment, because this was a better proxy for ddaD dendrite folding. To compare neurons in animals crawling at different speeds, the time window and the amplitude of each trace were normalized and interpolated across events (see STAR Methods). (E) SCAPE imaging of a *GMR10D05-Gal4, UAS-CD4tdGFP* larva during crawling showing dorsal cluster dendrite dynamics. Yellow box indicates neurons examined in the time-lapse sequences below. (E′-E‴) Dorsal (E′) and side (E″) views in which dmd1 soma and dendrite bundle are traced with a dashed yellow line; tracing is shown in (E‴). dbd is noted with an arrowhead. (F) Schematic and inset image showing neurons imaged together in (E). Inset: pseudo-colored neurons; ddaE (blue), ddaD (green), dbd (yellow), and dmd1 (pink). The dashed line represents the outline of ddaE and ddaD dendritic fields. (G and H) Mean (± SD) calcium response (solid line) of dmd1 (G) and dbd (H) soma during segment contraction (quantification and representation are the same as in C above). (I–I″) SCAPE imaging of a *1129-Gal4, 20XUAS-IVS-GCaMP6f* (×2), *UAS-CD4-tdTomato* larva ventral side during crawling. The yellow box indicates the vbd neuron examined in (I′) and (I‴) ventral and side views. (J) Mean (± SD) calcium response (solid line) of vbd soma during segment contraction (quantification and representation are the same as in C above). For (A), (E), and (I), images show representative SCAPE MIPs over an 80- to 95-μm-depth range from a 160- to 165-μm-deep volume (to exclude gut auto-fluorescence; square root grayscale). See Video S4 for dendrite motion dynamics and Video S5 for GCaMP dynamics related to (A), (E), and (I). Sample sizes:(c) 3 animals, n = 10 cells, 17 events; (D) 3 animals, n = 7 cells, 11 events; (G and H) n = 4 animals, n = 8 cells, 16 events; (J) n = 4 animals, n = 14 cells, 14 events. Figure S2 shows GCaMP and tdTomato images and single-neuron GCaMP activity from all genotypes. All ribbons represent SD. Posterior is to the left for all images. Scale bars, 100 μm (A, E, and I) and 50 μm (A′, A″, E′, E″, F, I′, and I″).

**Figure 4.**
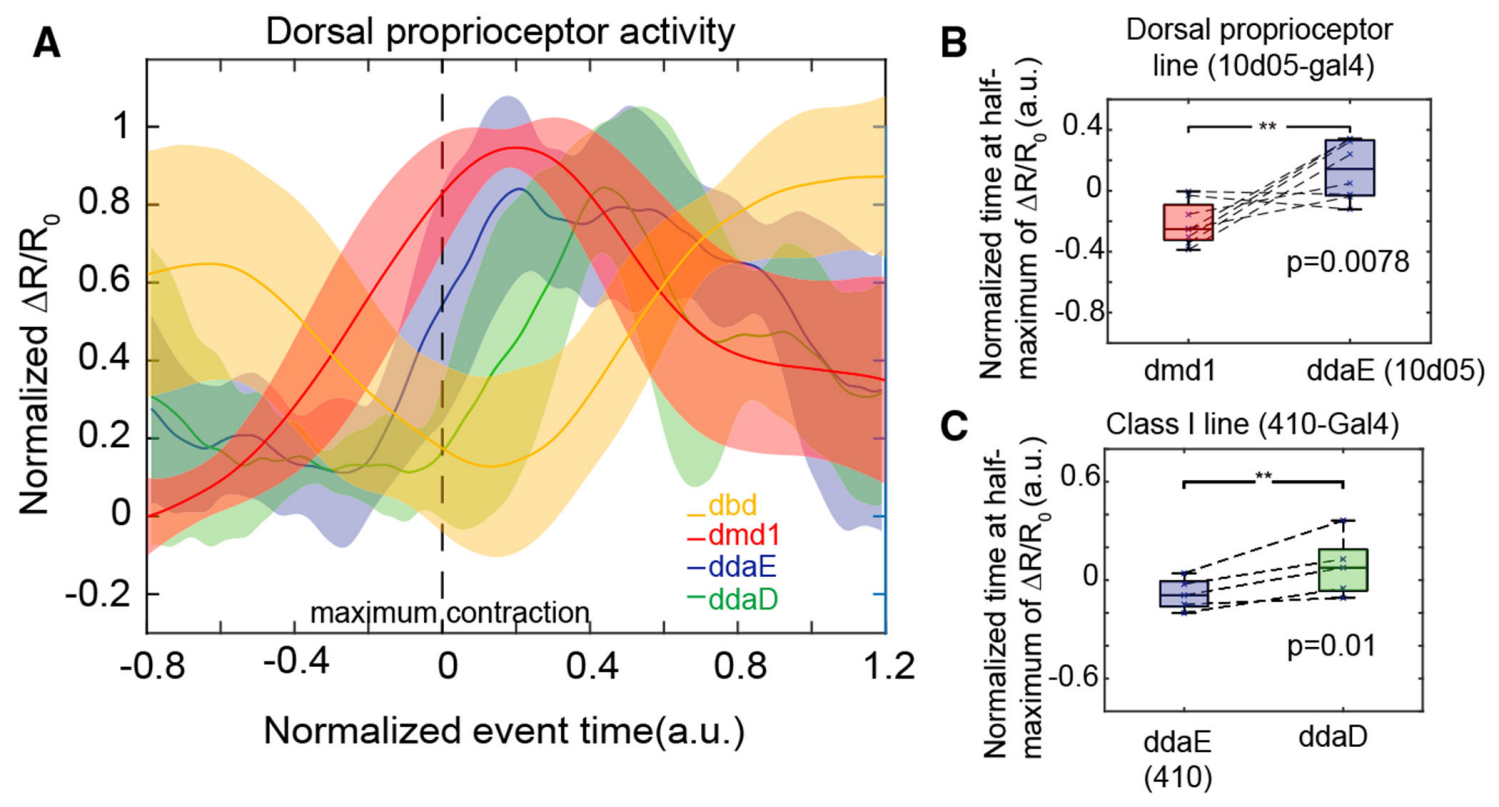
Each Dorsal Proprioceptor Type Is Activated Sequentially during Segment Contraction (A) Mean calcium response (± SD) of dbd, dmd1, ddaE, and ddaD during segment contraction. dmd1 and dbd data are the same as shown in Figures 3G and 3H. This plot includes a subset of ddaE and ddaD activity data shown in Figures 3C and 3D from paired cells within a segment (n = 4 animals, 5 cells, 5 events). Data are aligned using the time at maximum contraction (as measured by the distance between ddaE and homologous ddaE in the posterior segment), which is set at t = 0 s for each event. The time window and the amplitude of ΔR/R_0_ of each trace were normalized and interpolated across events (see STAR Methods). (B) To test the lag between dmd1 and ddaE activity, we compared the time at half-maximum calcium activity from paired cells within a segment in a GMR*10D05-Gal4, 20XUAS-IVS-GCaMP6f* (×2), *UAS-CD4-tdTomato* larva (n = 4 animals, n = 8 cells, 8 events). ddaE activity occurs significantly later than dmd1 activity (p = 0.0078) by single-tailed paired t test. (C) To test the lag between ddaE and ddaD, we compared the time at half-maximum calcium activity from *410-Gal4, 20XUAS-IVS-GCaMP6f* (×2), *UAS-CD4-tdTomato* animals (n = 4 animals, n = 5 cells, 5 events). Data are from the same cell pairs as analyzed in (A). ddaD activity occurs significantly later than ddaE activity (p = 0.01) by single-tailed paired t test.

Imaging of dorsal class I neurons revealed that ddaE and ddaD dendrites deform as the peristaltic wave enters each segment, and flatten as the wave passes (Figures 3A–3A‴; Video S4, first section). Although there is some variability in the degree of dendrite deformation, we consistently see folding in both cell types, with ddaE folding before ddaD (Figures 3A″, 3A‴, and 3B) in synchrony with the posterior-to-anterior progression of peristaltic waves. Like vpda, calcium dynamics revealed increases in dorsal class I activity during segment contraction (Figures 3C, 3D, and S2; Video S5, first section). When comparing paired ddaE and ddaD cells within the same segment, responses of posterior ddaE and anterior ddaD neurons occurred in succession during segment contraction, with ddaE responding just before ddaD (Figures 4A and 4C), corresponding to the lag in dendrite folding. These data suggest that cellular calcium activity is a result of dendritic folding in all class I neurons. As a control, larvae were imaged under compression with a glass coverslip that prevented physical folding of the ddaD dendrites (Figure S3). In this case, no increases in ddaD calcium activity were seen during forward crawling, consistent with dendritic folding driving calcium activity. This same compression did not prevent dendritic folding in ddaE neurons, and accordingly this cell type continued to show activity during crawling. This result further highlights the importance of imaging freely crawling larvae for characterization of locomotion, because physical restraint itself appears to influence proprioceptive signaling.

The remaining three proprioceptor types have relatively internal locations and complex 3D motion paths during crawling. We leveraged SCAPE’s high-speed volumetric imaging capabilities to capture the distinct 3D movements and activity dynamics of these dmd1, dbd, and vbd proprioceptors during crawling. dmd1 dendrites were slack and coiled prior to the contraction wave, then, as the segment contracted, the dendrite bundle stretched anteriorly and was then pulled deeper into the animal (Figures 3E′–3E‴; Video S4, second section). As the segment extended, the bundle swung posteriorly and then returned to the coiled position. GCaMP imaging revealed increases in calcium activity in dmd1 during segment contraction, as the dendrite bundle stretched (Figures 3G and S2; Video S5, second section).

dbd and vbd dendrites folded as the segment contracted (Figure 3E′, arrowheads; Figures 3I–3I″; Video S4). For dbd, GCaMP fluorescence peaked during segment stretch (Figures 3H and S2; Video S5, second section), consistent with previous electrophysiology results in a dissected preparation [22]. However, in contrast to dbd, GCaMP fluorescence in vbd peaked during segment contraction (Figures 3J and S2; Video S5, third section). Thus, two proprioceptors with similar morphologies and dendrite dynamics can show distinct responses during crawling.

We next tracked groups of co-labeled dorsal proprioceptors to directly compare the timing of their activity during forward crawling. We found that each cell type is active sequentially during segment contraction (Figure 4). dbd is most active in a stretched or relaxed segment. Then, as the segment contracts, dmd1 activity increases first, followed by the class I neurons (ddaE and ddaD) as the cuticle folds during segment shortening (Figure 4A). Comparison of the time at half-maximum calcium activity confirmed that ddaD activity was significantly delayed relative to ddaE during forward crawling (Figure 4C; p = 0.01 by single-tail paired t test) and that ddaE activity occurs significantly later than dmd1 activity during forward crawling (Figure 4B; p = 0.0078 by single-tail paired t test). These data suggest that the unique dendrite morphology of each dorsal proprioceptor tunes each cell to respond at different times during a peristaltic wave. These activities could provide a continuum of cell-type-specific encoding during movement.

### Proprioceptor Activity Can Simultaneously Code for Head Turning and Retraction

In addition to imaging simple forward crawling, our SCAPE imaging experiments captured a wide range of movements during exploration. To examine how proprioceptive activity might provide feedback during more complex body movements, we mounted the larvae in a small arena bounded by agarose. With this setup, we were able to track and extract dorsal sensory activity during exploratory head movements.

**Figure 5.**
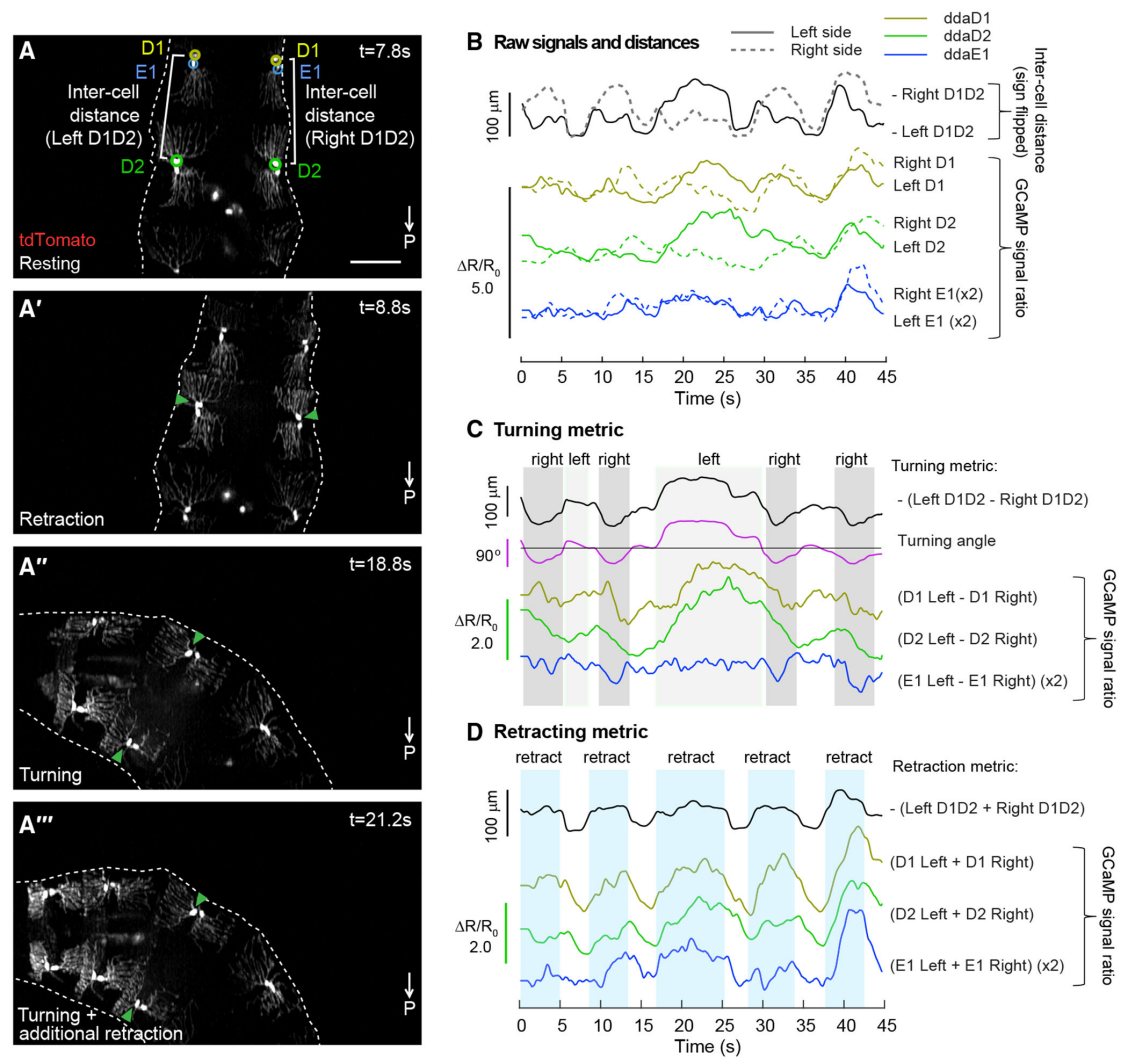
Dorsal Proprioceptor Activity Can Simultaneously Code for Head Turning and Retraction (A–A‴) SCAPE imaging of dorsal class I neurons labeled with *410-Gal4, 20XUAS-IVS-GCaMP6f* (x2), *UAS-CD4-tdTomato*, during exploratory behavior. Posterior is to the bottom of all images. tdTomato channel is shown. MIP over an 80-μm-depth range from a 140-μm-deep volume (square root grayscale). GCaMP fluorescence was quantified from circled neurons, ddaD (D1, yellow-green) and ddaE (E1, blue) in segment T3, and ddaD (D2, green) in segment A1. Inter-cell distance (white brackets) is quantified as the distance between ipsilateral D1 and D2. Representative behaviors are shown including resting (A), retraction only (A′), and turning (A″ and A‴). Turning showed different levels of retraction (e.g., A‴ shows more retraction than A″). Video S6 shows this dataset. (B) Calcium activity (measured as the ratio of GCaMP to tdTomato fluorescence) in left (solid) and right (dashed) D1, D2, and E1 neurons is compared to inter-cell distance (black). The sign is flipped on inter-cell distance measurements, so larger values represent shorter distances. ddaE activity was smaller than ddaD activity, so E1 data are shown at 2×. See Data S1 for correlation values. (C) Plots of the difference in calcium activity between contralateral cells compared to the difference in contralateral inter-cell distances (our turning metric) and the calculated angle of the D1 segment. (D) Plots of the sum of calcium activity between contralateral cells and the sum of contralateral inter-cell distances (our retraction metric). Scale bar, 100 μm.

In many cases, we observed typical exploration behavior, in which larvae bend their bodies asymmetrically to turn left or right, while concurrently retracting the head (Figures 5A–5A‴; Video S6). These behaviors are not always synchronized, which can lead to a complex motion path for each neuron and segment compared to locomotion. However, SCAPE recordings confirmed that even for this less stereotyped motion, class I activity increases continue to be associated with segment contraction. In the dataset shown in Figure 5, we focused on ddaD and ddaE within thoracic segment T3 (termed D1 and E1), and ddaD within abdominal segment A1 (termed D2), because these cells were deformed by the observed exploratory movements. Calcium activity in these cells correlated with the distance between ipsilateral D1 and D2 neurons (termed inter-cell distance) (Figure 5B; Video S6; see Data S1 for correlation coefficients and further discussion of ddaE). Calcium signal changes in ddaD neurons were greater than those in ddaE, consistent with greater measured changes in dendrite length for ddaD than for ddaE neurons during these turning and retraction events (ddaD, 32% ± 25% SD; ddaE, 10% ± 6.6% SD).

We next examined whether the seemingly complex inter-cell distances and calcium signals could be interpreted in terms of the larva’s combined turning and head retracting behavior. Examining the physical properties of the larva’s motion (see geometric model in STAR Methods), we note that head retraction and turning are distinct in that retraction is symmetric and head turning is asymmetric. Subtracting the D1-D2 inter-cell distance on the left side of the larva from the D1-D2 inter-cell distance on the right-side neuron pair should thus cancel out the effect of symmetric head retraction. This differential distance was found to closely match the calculated angle of the segment (providing a turning metric). Furthermore, assuming rigid coupling between the left and right cells, we found that adding the left D1-D2 inter-cell distance to the right D1-D2 inter-cell distance cancelled out the effect of turning (which has a reciprocal effect on left and right inter-cell distance), providing a measure consistent with the timing of the larva’s head retractions (a retraction metric).

Following the same logic, we computed the difference and summation of GCaMP6f signals extracted from proprioceptors on the left and right sides of the body. Remarkably, we found that the difference between calcium signals on the left versus right side correlates well with turning angle, especially for ddaD cells (Figure 5C; Data S1). Similarly, the sum of the calcium signals from the left and the right sides correlates strongly with our retraction metric for each cell type (D1, E1, D2) (Figure 5D; Data S1). Correlation values are even stronger when delays between movement and calcium transients are incorporated into comparisons (Data S1).

These data indicate that proprioceptor activity during exploration can represent simultaneous head turning and retraction. Furthermore, our data suggest that linear combinations of calcium signals from left and right neuron pairs can provide independent metrics of turning and head retraction.

## DISCUSSION

This study demonstrates a new approach for live volumetric imaging of sensory activity in behaving animals, leveraging an optimized form of high-speed SCAPE microscopy [9, 10]. We used this methodology to examine the activity patterns of a heterogeneous collection of proprioceptive neurons during crawling, as well as during more complex movements such as head turning and retraction, to determine how larvae sense body-shape dynamics. Imaging revealed 3D distortion of proprioceptive dendrites during movement and GCaMP activity that occurred coincident with dendritic deformations. We note that our results are consistent with a complementary study by He et al., published in this issue of *Current Biology*, which examined ddaD and ddaE dorsal proprioceptors and also demonstrated increased activity during dendrite folding [24]. Their study elucidated that this deformation-dependent signaling is reliant on the mechanosensory channel TMC.

Our survey of the full set of hypothesized multidendritic proprioceptors in behaving larvae revealed that most neurons (all class I neurons, dmd1, and vbd) increase activity during segment contraction. By contrast, dbd neurons showed increased activity during segment stretch, which is consistent with previous electrophysiological recordings of dbd in a dissected preparation [22]. The temporal precision afforded by high-speed SCAPE microscopy further revealed that different proprioceptors exhibit sequential onset of activity during forward crawling. Timing of activity was associated with distinct dendrite morphologies and movement dynamics, suggesting that proprioceptors monitor different features of segment deformation. The complementary sensing of segment contraction versus stretch in class I, dmd1, and vbd versus dbd neurons provides an additional measure of movement that is conceptually similar to the responses of Golgi tendon organs versus muscle spindles in mammals [25]. Combined, these results indicate that this set of proprioceptors function together to provide a continuum of sensory feedback describing the diverse 3D dynamics of the larval body.

Prior work suggested that the proprioceptors studied here have partially redundant functions during forward crawling because silencing different subsets caused similar behavioral deficits, namely slower crawling, whereas silencing both subsets had a more severe effect [14]. Slow locomotion may be a common outcome in a larva that is lacking in part of its sensory feedback circuit, yet our results suggest that each cell type has a unique role. Our demonstration of the varying activity dynamics of proprioceptors during crawling and more complex movements indicates that diverse sensory information is available to the larva, and suggests that feedback from a combination of these sensors could be used to infer aspects of speed, angle, restraint, and overall body deformation. This feedback system is likely to be important for a wide range of complex behaviors, such as body bending and nociceptive escape.

How can an understanding of proprioceptor activity patterns inform models of sensory feedback during locomotion? Electron microscopic reconstruction has shown that ddaD, vbd, and dmd1 proprioceptors synapse onto inhibitory premotor neurons (*period*-positive median segmental interneruons, A02b) [26], which promote segment relaxation and anterior wave propagation [27]. Thus, activity of these sensory neurons may signal successful segment contraction and promote forward locomotion, in part by promoting segment relaxation. Furthermore, vpda neurons provide input onto excitatory premotor neurons A27h, which acts through GABAergic dorsolateral (GDL) interneurons to inhibit contraction in neighboring anterior segments, thereby preventing premature wave propagation [28]. In this way, vpda feedback could contribute to proper timing of contraction in anterior segments during forward crawling. In contrast to other proprioceptors, dbd neurons are active during segment stretch. Their connectivity also tends to segregate from contraction-sensing neurons [26, 29], and understanding how the timing of this input promotes wave propagation is an important future question. Our dynamic recordings of the function of these neurons during not just crawling but also exploration behavior provide essential new boundary data for testing putative network models derived from this anatomical roadmap.

SCAPE’s high-speed 3D imaging capabilities enabled 10 VPS imaging of larvae during rapid locomotion. Fast volumetric imaging not only prevented motion artifacts but also revealed both the 3D motion dynamics and cellular activity associated with crawling behavior. SCAPE’s large, 1-mm-wide field of view allowed multiple cells along the larva to be monitored at once, while providing sufficient resolution to identify individual dendrite branches. Because SCAPE data are truly 3D, dynamics could be examined in any section or view. Additionally, fast two-color imaging enabled simultaneous 3D tracking of cells, monitoring of GCaMP activity, and correction for motion-related intensity effects. Our demonstration that larvae that are compressed during crawling exhibit altered dendrite deformation, and thus altered proprioceptive signaling (Figure S3), underscores the benefit of being able to image unconstrained larvae, volumetrically, in real time. Furthermore, rapid volumetric imaging allowed for the analysis of sensory responses during non-stereotyped, exploratory head movements in 3 dimensions, revealing activity patterns that could be utilized for encoding of complex, simultaneous movements. This finding also demonstrates the quantitative nature of SCAPE data and its high signal to noise, which enabled real-time imaging of neural responses without averaging from multiple neurons.

Here, we provide an example of how high-resolution, high-speed volumetric imaging enabled investigation of the previously intractable question of how different types of proprioceptive neurons encode forward locomotion and exploration behavior during naturalistic movement. Imaging could readily be extended to explore a wider range of locomotor behaviors such as escape behavior, in addition to other sensory modalities such as gustation and olfaction. Detectable signals reveal rich details including the firing dynamics of dendrites and axonal projections during crawling. Waves of activity in central neurons within the ventral nerve cord can also be observed. We expect that the *in vivo* SCAPE microscopy platform utilized here could ultimately allow complete activity mapping of sensory activity during naturalistic behaviors throughout the larval CNS. Using SCAPE, it is conceivable to assess how activity from proprioceptive neurons modulates central circuits that execute motor outputs, which will provide critical information for a dissection of the neural control of behavior with whole-animal resolution.

## STAR★METHODS

### CONTACT FOR REAGENT AND RESOURCE SHARING

Further information and requests for resources should be directed to and will be fulfilled by the Lead Contact, Elizabeth Hillman (eh2245@columbia.edu).

### EXPERIMENTAL MODEL AND SUBJECT DETAILS

*Drosophila melanogaster* strains were maintained on standard molasses food (agar, cornmeal, yeast, molasses, methylparaben, ethanol, propionic acid, penicillin, streptomycin) at 25°C, 60% humidity. Fly lines were obtained from the Bloomington (BL) *Drosophila* Stock Center or published sources as noted below. To image class I da neurons we used *221-Gal4* [30], *UAS CD4*-tdGFP (BL#35839) and *IT.410-Gal4* (BL#63298), *20XUAS-IVS-GCaMP6f* (2 copies, BL#52869 and BL#42747), *UAS-CD4-tdTomato* (BL#35841). To image GFP dynamics as a control, we used *IT.410-Gal4, 20XUAS mCD8::GFP* (BL#32194), *UAS-CD4-tdTomato.* To image all dorsal proprioceptors, we used GMR*10D05-Gal4* (BL#48438), *20XUAS mCD8::GFP* or GMR*10D05-Gal4, 20XUAS-IVS-GCaMP6f (2 copies), UAS-CD4-tdTomato.* To image vbd we used *IT.1129-Gal4* (BL#65461), *20XUAS-IVS-GCaMP6f (2 copies), UAS-CD4-tdTomato.* Animals of either sex were used. 2^nd^ instar larvae were used for all imaging experiments, except 3^rd^ instar larvae were used for imaging of compressed animals.

### METHOD DETAILS

#### SCAPE Image acquisition

High-speed volumetric imaging of crawling larvae was performed using a custom swept confocally aligned planar excitation (SCAPE) microscope extended from designs described in [9]. Briefly, high speed 3D imaging is achieved by illuminating the sample with an oblique light sheet through a high NA objective lens [31]. Fluorescence light excited by this sheet (extending in y-z’) is collected by the same objective lens (in this case an Olympus XLUMPLFLN 20XW 1.0 NA water immersion objective with a 2mm working distance). A galvanometer mirror in the system is positioned to both cause the oblique light sheet to scan from side to side across the sample (in the x direction, without a change in the angle of the sheet) but also to descan returning fluorescence light. This optical path results in an intermediate, descanned oblique image plane which is stationary yet always co-aligned with the plane in the sample that is being illuminated by the scanning light sheet. Image rotation optics and a fast sCMOS camera (Andor Zyla 4.2) are then focused to capture these y-z’ images at over 1000 frames per second as the sheet is scanned in the sample in the x direction. Data is then reshaped into a 3D volume by stacking successive y-z’ planes according to the scanning mirror’s x-position. All other system parts including the objective and sample stage are stationary during high speed 3D image acquisition, including the primary objective lens and sample stage. No image reconstruction procedures besides correction for the sheet’s oblique angle were used for data shown in this study.

For SCAPE imaging in this study, numerous refinements to our original SCAPE design were made to improve resolution and field of view, including optimization of light sheet formation to achieve better uniformity. The scanning system was optimized to maximize the system’s field of view, while optimized alignment of the detection system reduced aberrations and increased numerical aperture to yield higher diffraction limited resolution and light throughput. The system’s stationary objective was configured in an inverted arrangement for the ventral side imaging and an upright arrangement for the dorsal side imaging. Dual-color imaging was achieved using a custom-built dual color image splitter in front of the sCMOS camera. 488 nm excitation (< 5 mW at the sample, Coherent OBIS) was used to excite fluorescence in both channels, with 525/45 nm and 600/50 nm emission filters in the green and red emission channels respectively. The system’s camera frame rate to read 150-200 rows (corresponding to oblique depths along z’) was 1000-1300Hz, with an x-scanning step size of 2~3 μm to achieve 10 volumes per second imaging over a field of view of 1000 × 250 × 195 μm (y-x-z)(scan parameters varied for different trials, based on the size difference of each larva).

To image forward crawling, 2nd instar larvae were chosen to image multiple segments at once. Larvae were imaged at room temperature while positioned within a 300 μm wide water-filled channel bounded by FEP spacers and covered by a 40mm × 24mm cover glass. When imaging the ventral side, the channel was positioned on a 50mm × 24mm cover glass, when imaging the dorsal side, the channel was positioned on a glass slide. Each trial acquired data for up to 120 s or was terminated earlier if the larva crawled to the end of the channel. A manual translation stage aligned along the FEP channel axis was used to keep the larva in the field of view during the acquisition as needed.

To image compressed animals, we reproduced typical conditions for confocal microscopy imaging of larvae. As such, 3rd instar larvae were positioned in a 50:50 mixture of halocarbon oils 27 and 700 to enhance compression with an overlying coverslip. We ensured that cross sections of the animal showed body compression during imaging (Figure S3).

To image complex head movements (turns and retractions) during exploration behavior, we constrained 2^nd^ instar larvae in a small water-filled arena bounded by 10% agarose, with a coverslip on top. The size of the arena is about 1000 μm by 500 μm and it was made on a ~200 μm thick agarose pad.

All the functional calcium signal analysis used signals extracted from raw, linear-scale imaging data. However, for the visualizations of SCAPE images shown in the figures and movies, raw camera 16bit data was square root scaled to enhance the visible dynamic range to avoid display saturation and to make all components (soma and dendrites) more visible. Resulting pixel values are then shown on a linear gray, red or green colorscale without further adjustment. SCAPE data was interpolated to uniform voxels with spline smoothing for all the figures and movies, and sharpened using the imageJ function ‘unsharp mask’ (radius:1.5, weight:0.4) to enhance dendrite visualization except for Figures 2A and 2A′ and Videos S2, S3, and S4, fourth section, and S5, first and second sections. None of the raw SCAPE imaging data was saturated during acquisition. Images and movies were generated using MATLAB and ImageJ.

#### Cell tracking and ratiometric GCaMP analysis

For SCAPE imaging quantification, vpda, vbd, and dbd neurons were first manually selected and then automatically tracked in 3D space for the duration of the run that the neuron was within the field of view, using MATLAB. For other neuron types (ddaD, ddaE, dmd1), since the distance between neighboring neurons is small, automatic tracking was performed under supervision and manually corrected when needed. Tracked neurons are from segments A1-A6. This 3D tracking provided behavioral information related to the animal’s physical movements, as well as fiducials for extraction of GCaMP fluorescence from the cells during movement.

We chose GCaMP6f because it has a fast decay time (~400 ms) [32], which is faster than the decay seen in our data (see real time traces Figures 2, S1, and S2, decay happens over seconds). For fluorescence extraction, tracking regions of interest (ROIs) were defined as the smallest rectangular 3D cube around the tracked cell that encompassed the entire cell body. Average fluorescence intensity values of GCaMP6f and tdTomato were then extracted from these ROIs for each time point. A ratio between GCaMP6f and tdTomato was calculated after subtraction of background signal to account for the motion induced intensity change for each frame (yielding the green-to-red ratio R). The average of the lowest 10% ratio values was used as the baseline (R_0_) for each ROI. The GCaMP signal reported as neural activity at each time point then corresponds to the change in this ratio from baseline (ΔR/R_0_). To demonstrate this process, raw red, green and ratiometric signals are shown in Figure S1. In addition, control measurements are shown that applied the same analysis to larvae co-expressing tdTomato and static GFP. In the GFP case, dynamic changes in ΔR/R_0_ were insignificant, confirming the sensitivity of our (ΔR/R_0_) measure to the intracellular calcium-dependent fluorescence of GCaMP.

#### Calculating segment and dendrite dynamics

To relate calcium dynamics to segmental contraction and extension phases, changes in inter-cell distances were calculated from the tracked cell coordinates – defined as the distance between the measured neuron and a homologous neuron in the posterior or anterior segment over time. For vpda and vbd, we plotted posterior inter-cell distance between the measured neuron and a homologous neuron in the posterior segment. For dbd, dmd1, ddaE, and ddaD we plotted the posterior inter-cell distance between the ddaE neuron in the same segment as the cell of interest and the homologous neuron in the next posterior segment. This allowed us to directly compare the timing of dorsal neuron activity. For some plots of ddaD activity (Figures 3D and S2), we plotted anterior inter-cell distance between the measured neuron and the homologous ddaD neuron in the anterior segment, since this was a better proxy for dendrite folding.

To directly measure dendrite dynamics in ddaD and ddaE neurons during crawing (Figures 3B and S3) and turning (Figure 5), dendrite length was measured as a 180 degree line from the cell body to the furthest visible dendrite. In crawling animals (Figure 3B), 2-3 cells of each type from segments A1-A6 were analyzed from 4 different animals, and each cell was analyzed during a different peristaltic wave.

#### Averaging dynamics across larvae

Real-time traces of inter-cell distance and ΔR/R_0_ shown in Figures 2, S1, and S2, demonstrate the high signal to noise and repeatability of our observations. However, these traces also demonstrate that the speed of crawling can vary quite significantly between animals, in addition to the relative amplitude of (ΔR/R_0_). To provide the aggregate, average properties of neural activity against segment contraction, it was thus necessary to normalize these differences between animals. To calculate mean calcium responses (ΔR/R_0_) in relation to segment contraction, we normalized amplitude of responses across events to 1, so as not to bias the average values to cells that responded more strongly (Figures 2D and 3). To plot mean calcium activity (ΔR/R_0_), inter-cell distance, or dendrite measurements across animals with different crawling speeds, normalization was applied in time based on the full width at half maximum (FWHM) of the mean inter-cell distance of every contraction for each animal (Figures 3B–3D, 3G, 3H, 3J, and 4A). 1 A.U. ranges from 0.7-2.5 s. We excluded the contraction events which inter-cell distance did not return back to the resting length, and we did not include more than two events per neuron for the averaging analysis. To test the activity timing lag between neuron types (Figures 4B and 4C), we compared the normalized time at half-maximum calcium activity.

To characterize the resting and contraction phase activity in Figure S1C, we defined the contraction phase as 2 × FWHM window centered at the maximum contraction point and resting phase as the 0.5 × FWHM window prior to the contraction phase. The resting phase GFP-tdTomato or GCaMP6f-tdtomato ratio value was calculated by taking the mean ΔR/R_0_ along the resting phase window. And the contraction phase GFP-tdTomato or GCaMP6f-tdtomato ratio value was calculated by taking the maximum ΔR/R_0_ over the contraction phase window.

#### Motion model for exploring larva

We made the following assumptions based on the properties of motion of the tracked neurons D1L (left anterior ddaD), D1R (right anterior ddaD), D2L (left posterior ddaD) and D2R (right posterior ddaD) that:

1) The lava’s right and left neurons are positioned either side of a rigid bar of length ~2p
2) Turning motion is given by a time-varying rotation θ (t) of the anterior bar about the midpoint of the posterior bar.
3) The retraction motion of the larva was approximated as a time-varying distance S(t) between the midpoints of each bar.

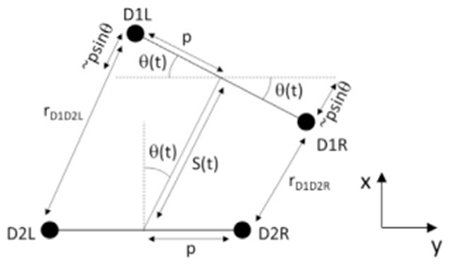
 From this construction, we can see that good approximations to the distance between D1R and D2R (r_D1D2R_) and D1L and D2L (r_D1D2L_) are simply:

(Equation 1) $$r_{D1D2L}=S_{2}(t)+p.\sin(q(t))\;r_{D1D2R}=S_{2}(t)-p.\sin(q(t))$$

From this relationship, it is simple to see that the subtraction or summation of these two inter-cell differences are going to yield:

(Equation 2) $$r_{D1D2L}-r_{D1D2R}=2p\sin q(t)$$

which is a pure function of turning angle, and

(Equation 3) $$r_{D1D2L}+r_{D1D2R}=2S_{2}(t)$$

which is a pure function of time-varying head retraction.

This ‘common mode rejection’ property holds true for the positional data of the larva’s neurons, as well as the subtraction and summation of the left and right GCaMP signal extracted from neurons innervating the inter-cell space.

A more rigorous derivation notes that the precise value of the inter-cell distance is given by:

(Equation 4) $$r_{D1D2L}=S_{2}(t)+p.\sin(q(t))+{\left({\left(p-p.\cos(q(t)\right)}^{2}+{\left(S_{2}(t)+p.\sin(q(t))\right)}^{2}\right)}^{1∕2}r_{D1D2R}=S_{2}(t)-p.\sin(q(t))+{\left({\left(p-p.\cos(q(t)\right)}^{2}+{\left(S_{2}(t)-p.\sin(q(t))\right)}^{2}\right)}^{1∕2}$$

however, the difference between this and our approximation is < 0.12% for the motion parameters of the larva observed.

### QUANTIFICATION AND STATISTICAL ANALYSIS

Statistical tests were performed using MATLAB. We evaluated the lag between dmd1 versus ddaE and ddaE versus ddaD activity by single tailed paired t test, alpha level 0.05. We evaluated the difference between the resting and contraction phases for the GFP-tdTomato and GCaMP6f-tdtomato ratios with two-tailed paired t test. No optimal sample-size estimation was calculated. Statistical parameters reported in figure legends. All p values are represented as: * < 0.05, ** < 0.01, and *** < 0.001.

### DATA AND SOFTWARE AVAILABILITY

Cell tracking, signal extraction and ratiometric correction was performed using custom MATLAB scripts that will be made available upon request. The data that support the findings of this study are available from the corresponding authors upon request.

## Supplementary Material

1

2

3

4

5

6

7

8

## Figures and Tables

**Table:** KEY RESOURCES TABLE

REAGENT or RESOURCE	SOURCE	IDENTIFIER
Experimental Models: Organisms/Strains		
w[*]; P{y[+t7.7] w[+mC] = 20XUAS-IVS-mCD8::GFP}attP2	Bloomington Drosophila Stock Center (BDSC)	BDSC: 32194
y[1] w[*]; P{w[+mC] = UAS-CD4-tdTom}7M1	BDSC	BDSC: 35841
y[1] w[*]; P{w[+mC] = UAS-CD4-tdGFP}8M2	BDSC	BDSC: 35839
w[1118]; PBac{y[+mDint2] w[+mC] = 20XUAS-IVS-GCaMP6f}VK00005	BDSC	BDSC: 52869
w[1118]; P{y[+t7.7] w[+mC] = 20XUAS-IVS-GCaMP6f}attP40	BDSC	BDSC: 42747
w[1118]; PBac{w[+mC] = IT.GAL4}Frl[1129-G4]	BDSC	BDSC: 65461
w[1118]; PBac{w[+mC] = IT.GAL4}CG14931[0410-G4]/CyO	BDSC	BDSC: 63298
w[1118]; P{y[+t7.7] w[+mC] = GMR10D05-GAL4}attP2	BDSC	BDSC: 48438
221-Gal4	[30]	Flybase ID: FBti0187661
Software and Algorithms		
Fiji	NIH	https://imagej.net/Fiji/Downloads
MATLAB	Mathworks	https://www.mathworks.com/
Solis	Andor	https://andor.oxinst.com/products/solis-software/

## Highlights

- High-speed, 3D SCAPE microscopy is used to image freely moving *Drosophila* larvae
- Calcium activity in proprioceptive neurons is coincident with dendrite deformation
- Proprioceptor subtypes show sequential activity patterns during forward crawling
- Sensors activate in different combinations during complex turns and retractions
