# Supplementary material for: Characterization of Proprioceptive System Dynamics in Behaving Drosophila Larvae Using High-Speed Volumetric Microscopy
Source: Curr Biol. Author manuscript; Available in PMC 2020 Mar 18. (PMC6624193; doi:10.1016/j.cub.2019.01.060)
Supplement: 2 [file NIHMS1522499-supplement-2.pdf]

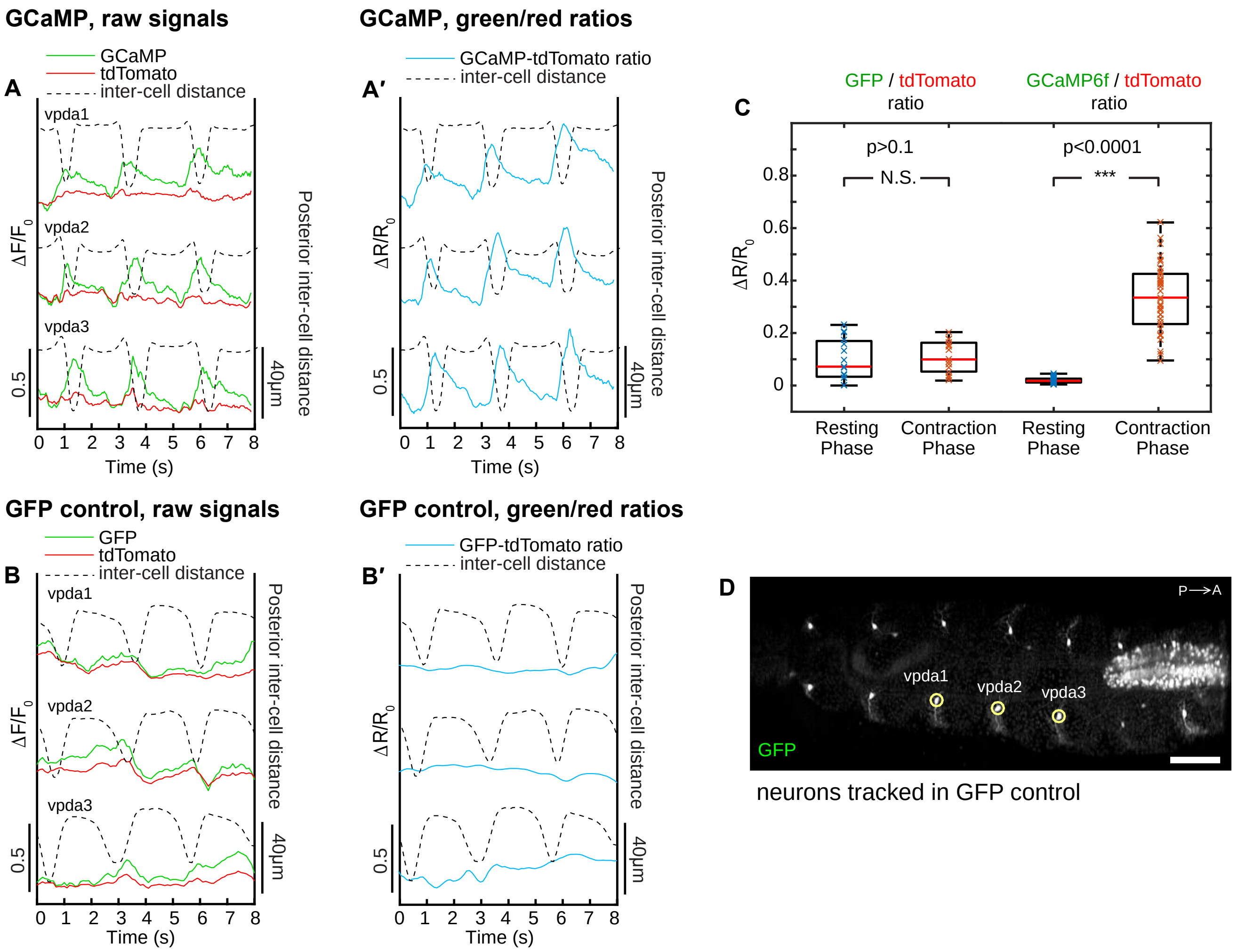

Figure S1. Ratiometrically measured calcium dynamics properly control for motion artifacts, related to Figure 2. (A) Change in fluorescence from baseline ($\Delta F/F_0$) in GCaMP6f (green) and tdTomato (red) during crawling in vpda neuron somas. Segment contraction is depicted with inter-cell distance (dashed lines). **(A´)** Change in ratio of GCaMP6f to tdTomato fluorescence ($\Delta R/R_0$, blue). Increases can be seen during segment contraction. **(B)** Change in fluorescence from baseline ($\Delta F/F_0$) in GFP (green) and tdTomato (red) during crawling in vpda neuron somas. Segment contraction is depicted with inter-cell distance (dashed lines). (**B**´) Change in ratio of GFP to tdTomato fluorescence ($\Delta R/R_0$, blue). No increase is associated with segment contraction. **(C)** Comparison of GFP-tdTomato and GCaMP6f-tdtomato ratios between resting and contraction phases (see methods). For GFP-tdTomato analysis, n= 2 animals, 7 cells, 14 events, for GCaMP6f-tdtomato analysis, n=3 animals, 22 cells, 26 events. Note that there is no difference between GFP-tdTomato ratios in the resting versus contraction phases, while there is a significant increase in GCaMP6f-tdTomato ratios during contraction ($p<0.001$, as measured by two-tailed t-test). **(D)** SCAPE imaging of *410-Gal4*, *20XUAS mCD8::GFP*, *UAS-CD4-tdTomato* animals during forward crawling, Ventral side. Imaging shows vpda neurons. GFP channel is shown. Posterior is to the left. Images are shown on a square root grayscale to reduce dynamic range for visualization of both cell bodies and dendrites. Scale bar=100µm.

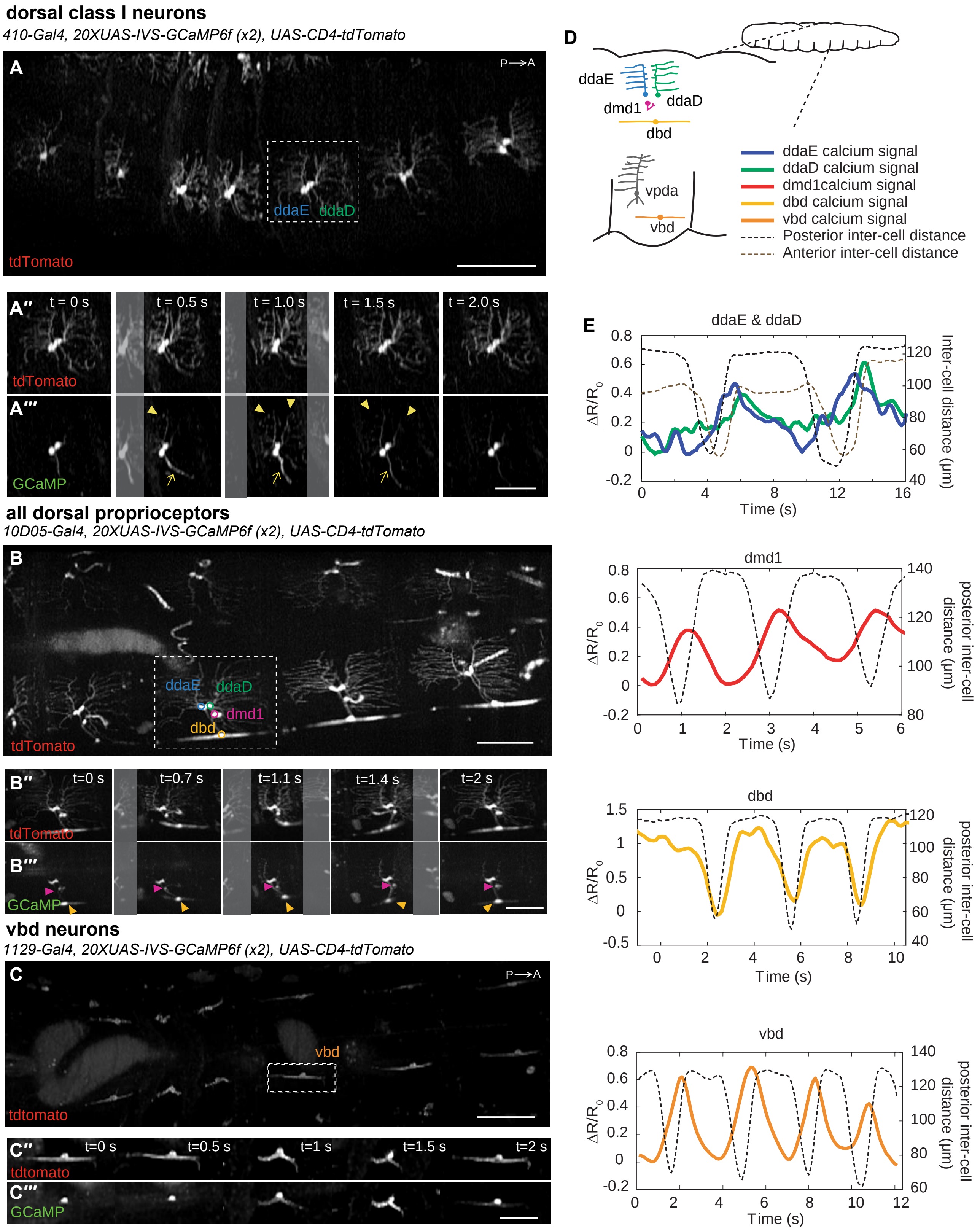

Figure S2. Examples of SCAPE imaging of GCaMP dynamics, related to Figure 3. Posterior is to the left for all images. For (**A-C**), images show representative SCAPE MIPs over a 35-90 μm depth range from a 160-200 deep volume (to exclude

gut autofluorescence, square root grayscale). Dashed box indicates neurons examined in time lapse sequences below, shown for both tdTomato and GCaMP channels. **(A-A´´´)** SCAPE imaging of *410-Gal4, 20XUAS-IVS-GCaMP6f* (x2), *UAS-CD4-tdTomato* larva. See Video S5, first section. Arrowheads indicate increases in dendritic GCaMP6f, arrows indicate increases in axon bundle (containing both ddaD and ddaE axons). Note ddaE dendrites are active before ddaD. **(B-B´´´)** SCAPE imaging of *GMR10D05-Gal4, 20XUAS-IVS-GCaMP6f* (x2), *UAS-CD4-tdTomato* larva. See Video S5, second section. Orange arrowhead marks dbd cell body, pink arrowhead marks dmd1 cell body. **(C-C´´´)** SCAPE imaging of *1129-Gal4, 20XUAS-IVS-GCaMP6f* (x2), *UAS-CD4-tdTomato* larva. See Video S5, third section. **(D)** Schematic of larval proprioceptive system. **(E)** Examples of single cell calcium activity dynamics during forward crawling. The calcium response is plotted in solid lines (quantified as $\Delta R/R_0$). The distance between the measured neuron and the posterior neuron (posterior inter-cell distance) is plotted in black dashed lines. The distance between the measured neuron and the anterior neuron (anterior inter-cell distance) is also plotted in brown dashed lines on the ddaD plot, since this is a better proxy for dendrite folding.

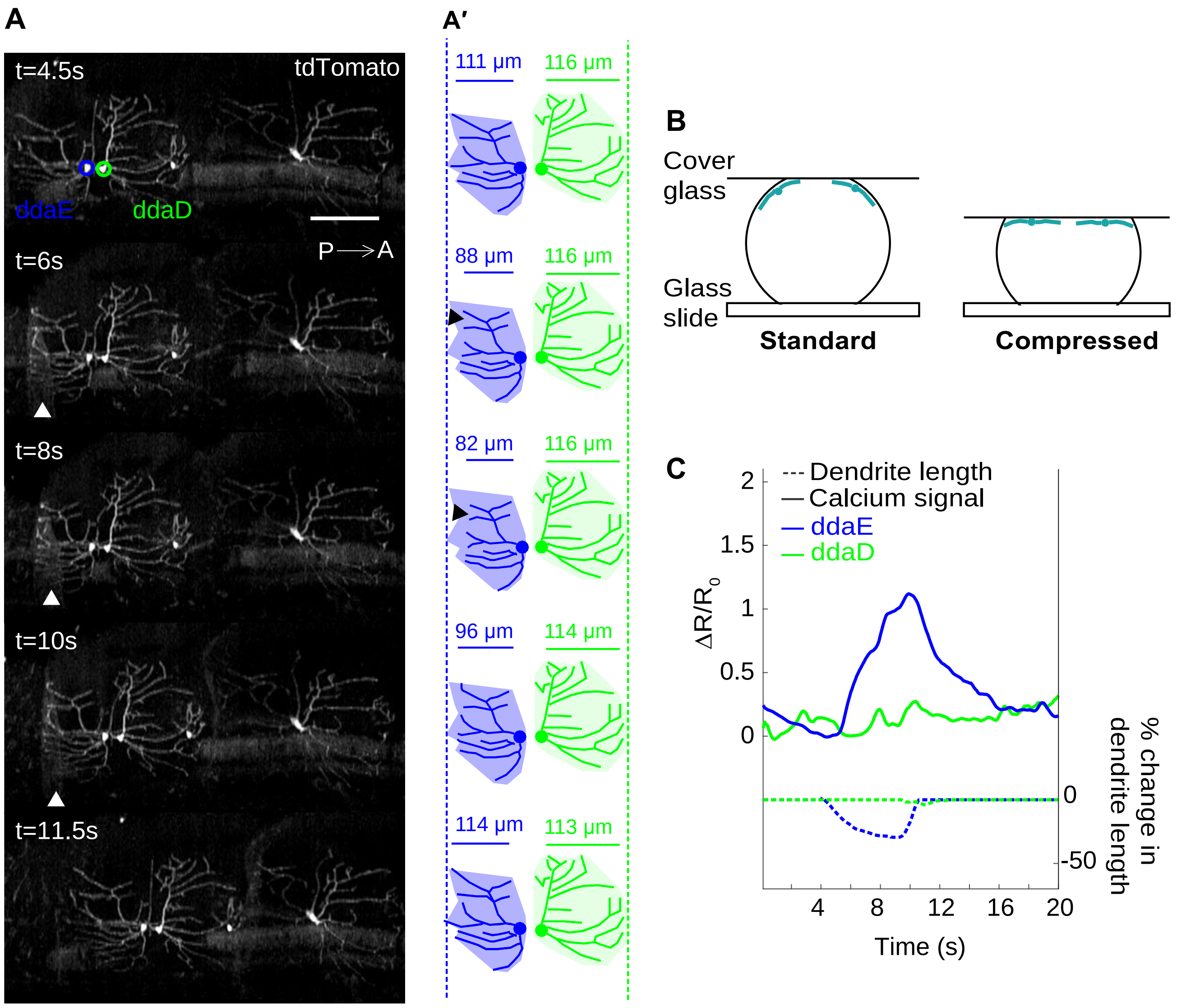

Figure S3. Sensory activity does not occur in the absence of dendritic folding, related to Figure 3.
(A) Time lapse of SCAPE imaging of dorsal class I neurons labeled with *410-Gal4*, *20XUAS-IVS-GCaMP6f* (x2), *UAS-CD4-tdTomato*, in a compressed preparation, which prevents dendritic folding in ddaD (see **(B)** and methods). TdTomato channel is shown to depict dendrite dynamics. Larva is 3rd instar. Posterior is to the left. (MIP) over a 50µm depth range from a 160µm deep volume. **(A´)** Tracing of time lapse data shown in **(A),** posterior cells. ddaE is blue and ddaD is green. Dotted lines and shaded areas represent extent of arbor in a relaxed segment. Measurements represent dendrite length (µm), a measure of dendrite folding. Arrows denote frames with dendrite folding. Note that ddaE dendrites fold, but not ddaD. **(B)** Schematic of compressed preparation. **(C)** Calcium responses ($\Delta R/R_0$, solid lines) and % change in dendrite length (dotted lines) in a compressed preparation of ddaE (blue) and ddaD (green) during segment contraction. Activity correlates with dendrite folding. Scale bar=100µm.
